# Supplementary material for: Coupling an Electroactive Pseudomonas putida KT2440 with Bioelectrochemical Rhamnolipid Production
Source: Microorganisms. 2020 Dec 10;8(12):1959. doi: 10.3390/microorganisms8121959 (PMC7763313; doi:10.3390/microorganisms8121959)
Supplement: Supplementary file 1 [file microorganisms-08-01959-s001.pdf]

Supplementary Materials

Coupling an electroactive *Pseudomonas putida* KT2440 with bioelectrochemical rhamnolipid production

Theresia D. Askitosari[1#], Carola Berger[2#], Till Tiso[1], Falk Harnisch[3], Lars M. Blank[1] and Miriam A. Rosenbaum[2,4*]

- # These authors contributed equally.
- 1 Institute of Applied Microbiology – iAMB, Aachen Biology and Biotechnology – ABBt, RWTH Aachen University, 52074 Aachen, Germany.
- 2 Department of Environmental Microbiology, Leibniz Institute for Natural Product Research and Infection Biology – Hans Knöll Institute, 07745 Jena, Germany.
- 3 Helmholtz Centre for Environmental Research – UFZ, 04318 Leipzig, Germany
- 4 Faculty of Biological Sciences, Friedrich Schiller University Jena, 07745 Jena, Germany.
- * Correspondence: miriam.rosenbaum@leibniz-hki.de; Tel.: +49-3641-532-1120

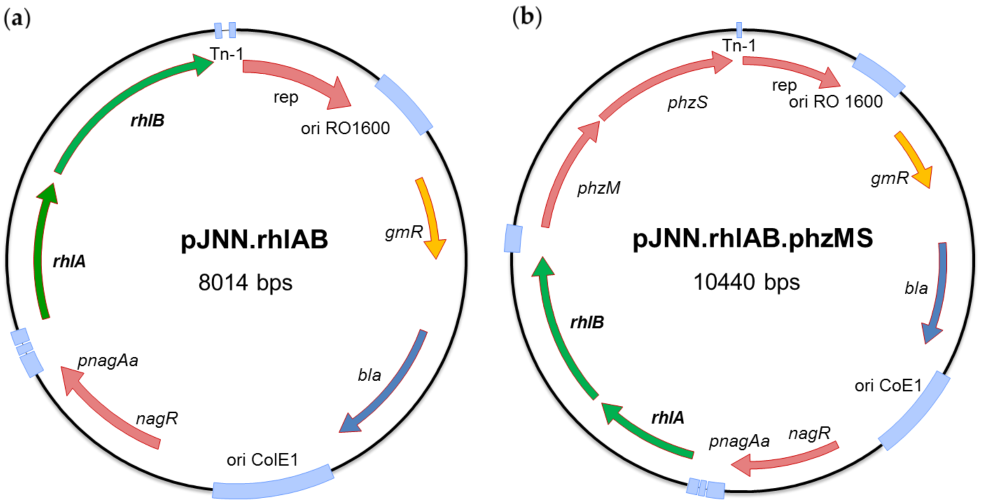

Figure S1: Constructed vectors expressing genes for mono-rhamnolipid- and phenazine synthesis. (a) pJNN.*rhlAB*, expressing the *rhlA* and *rhlB* genes from *P. aeruginosa* PAO1; (b) pJNN.*rhlAB.phzMS*, expressing the *rhlA, rhlB, phzM,* and *phzS* genes from *P. aeruginosa* PAO1. Both plasmids also contain the following elements: the gentamycin resistance cassette (gmR) for *Pseudomonas*, the ampicillin resistance cassette for *E.coli* (bla), the terminator (Tn-1), the origin of replication (*ori*RO1600 for *Pseudomonas* and *oriColE1* for *E.coli*), and the salycilate-induced promoter (*pnagAa*/*nagR*).

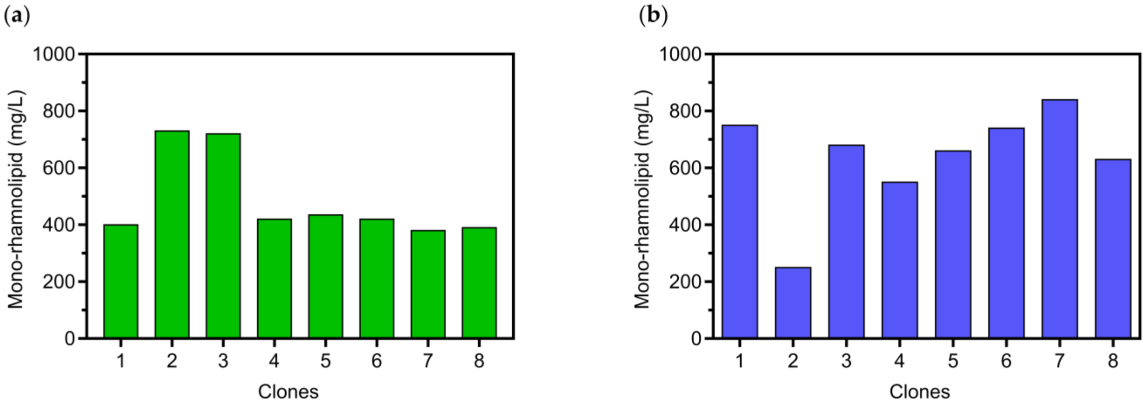

Figure S2: Heterologous rhamnolipid production of eight independent *P.putida* KT2440 clones cultivated in a micro-cultivation platform in LB media for 24 hours. (**a**) *P. putida* RL (carrying the pJNN.*rhlAB* plasmid); (**b**) *P. putida* RL-MS (carrying the pJNN.*rhlAB.phzMS* plasmid).

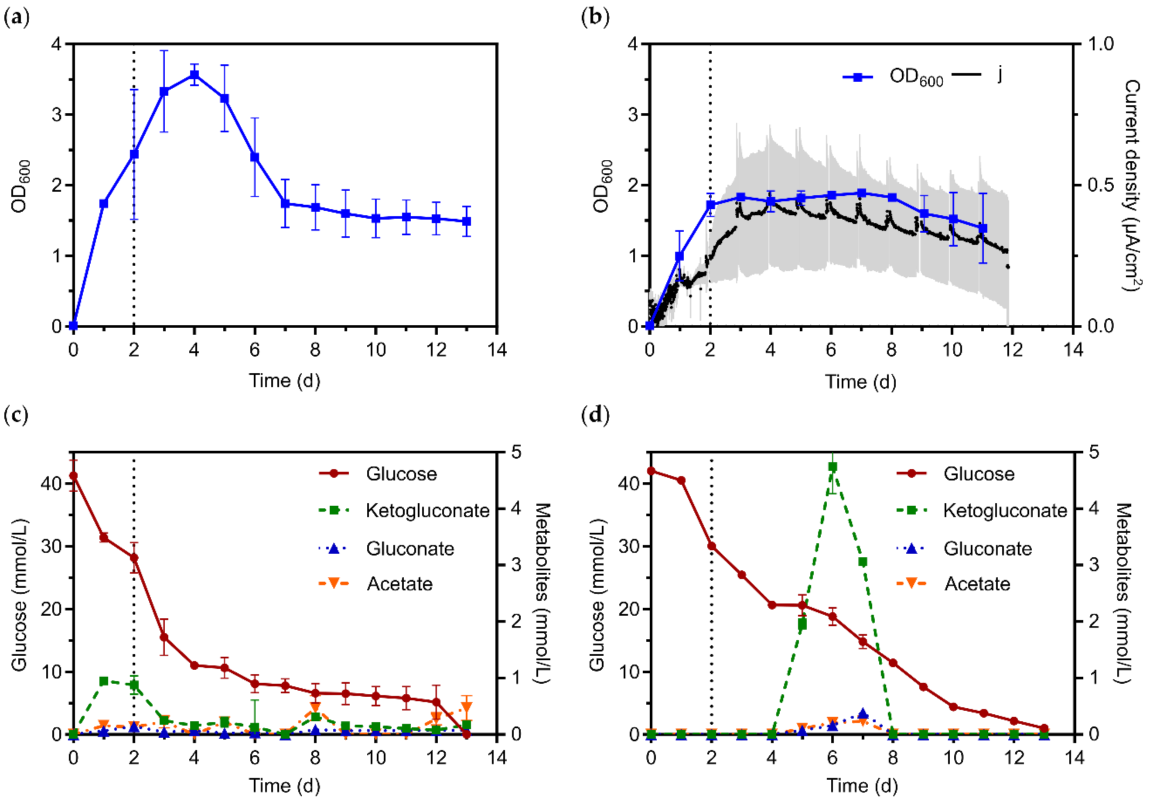

Figure S3: *P. putida* RL cultivation in 500-ml benchtop bioelectrochemical systems at open circuit ((**a**) & (**c**)) and with ((**b**) & (**d**)) an applied potential of 0.2 V (vs. Ag/AgCl). The reactors were actively aerated for the first 48 h with 30 mL/min followed by passive aeration of the headspace through open vent filters (dotted vertical line) (n=3). Rhamnolipids could not be detected.

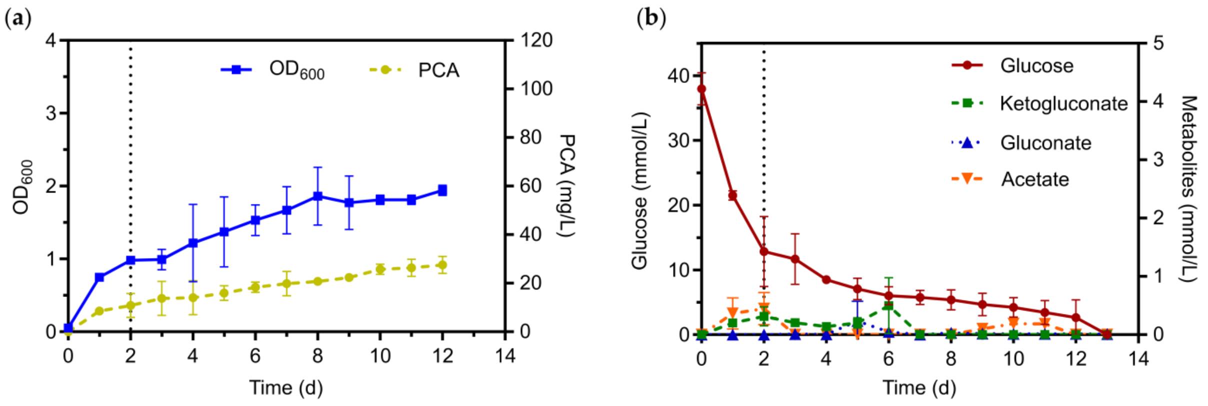

Figure S4: *P. putida* RL-PCA cultivation in 500-ml benchtop bioelectrochemical systems at open circuit potential. The reactors were actively aerated for the first 48 h with 30 mL/min followed by passive aeration of the headspace through open vent filters (dotted vertical line) (n=3). Rhamnolipids could not be detected.

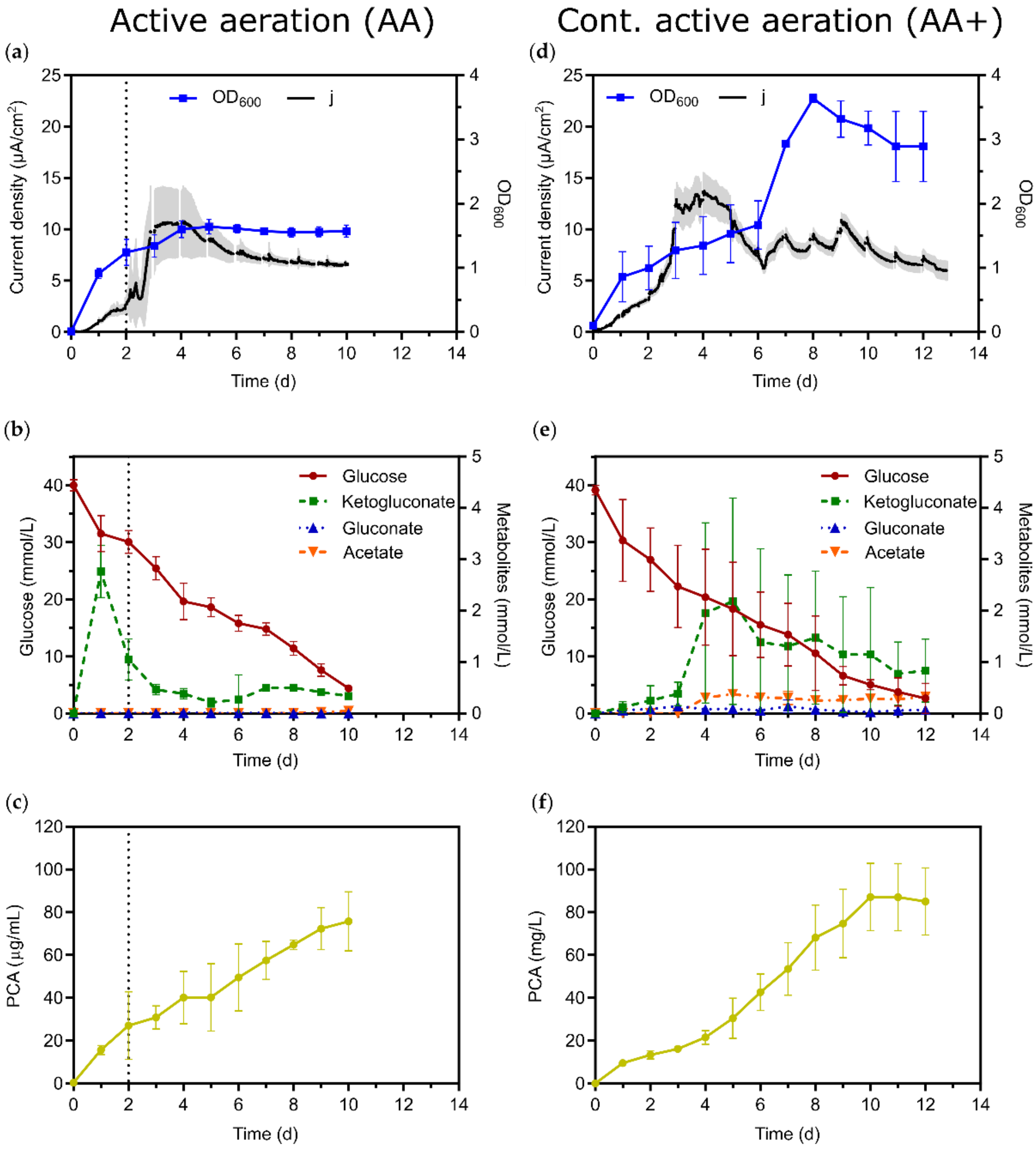

Figure S5: *P. putida* RL-PCA cultivations in 500-ml benchtop bioelectrochemical systems at an applied potential of 0.2 V (vs. Ag/AgCl). (**a**)-(**c**): The reactors were actively aerated for the first 48 h with 30 mL/min followed by passive aeration of the headspace through open vent filters (dotted vertical line) (n=3). (**d**)-(**e**) The reactors were actively aerated throughout the entire experiment at a flow rate of 50 mL/min (n=3). Rhamnolipids could not be detected.

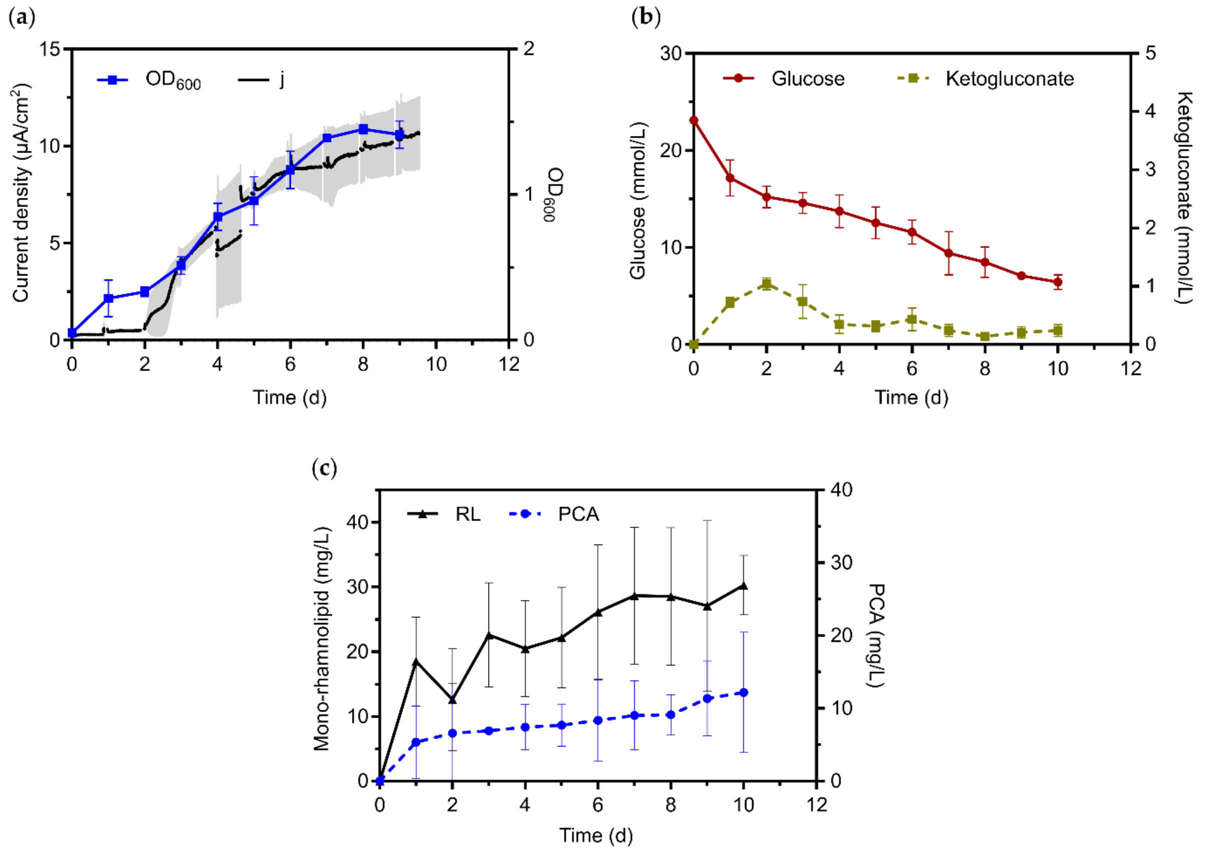

Figure S6: Repetition of passively aerated 500-ml benchtop bioelectrochemical systems of *P. putida* RL-PCA at an applied potential of 0.2 V (n=3), showing data for cell density (OD_{600}) and current production (**a**), glucose consumption and 2-ketogluconate production (**b**), formation of PCA and rhamnolipids (RL) (**c**).

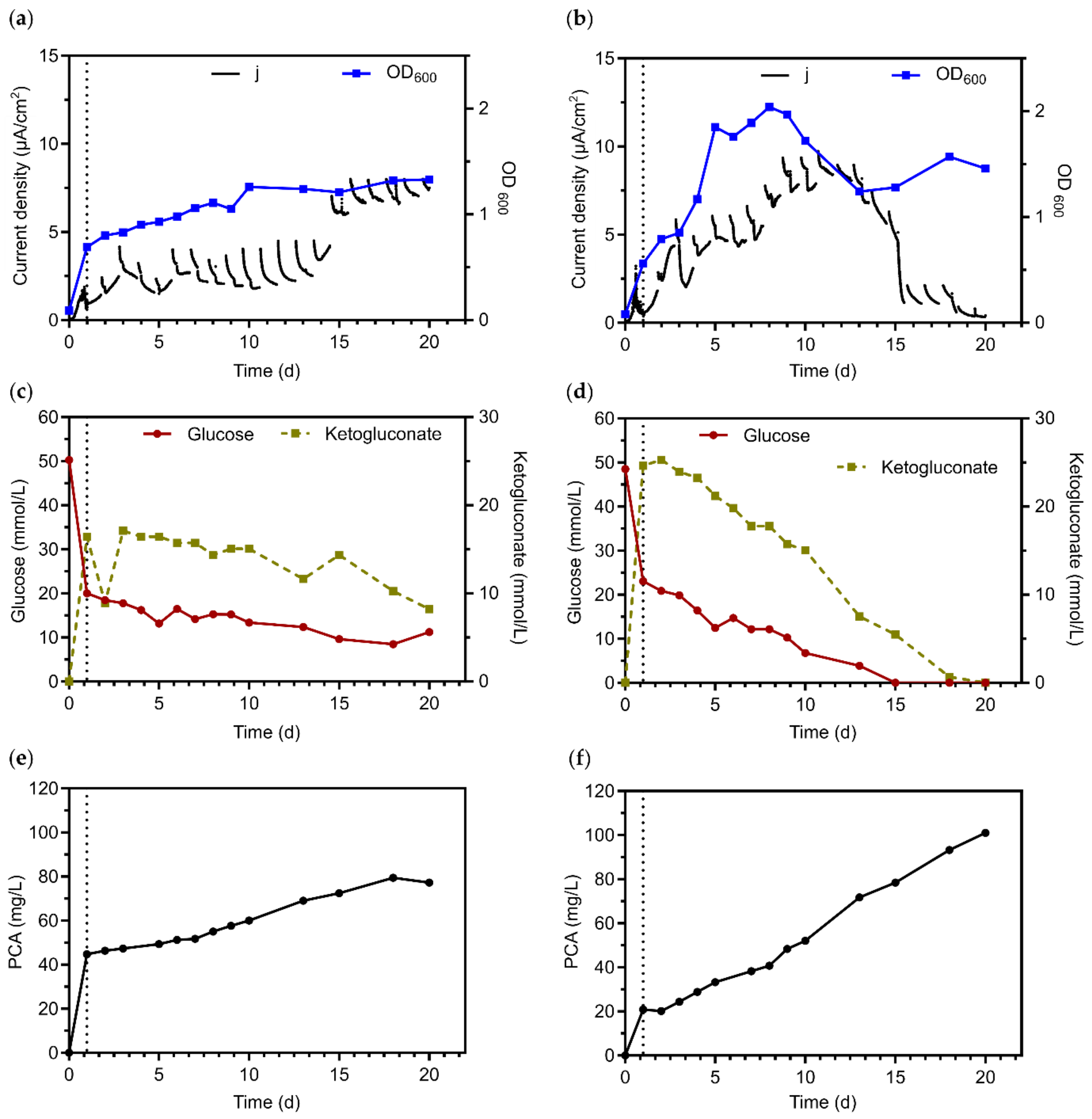

Figure S7: Duplicate bioelectrochemical systems 1-L electrobioreactor of *P. putida* RL-PCA at an applied potential of 0.2 V, showing data for cell density (OD_{600}) and current production ((**a**) & (**b**)), glucose consumption and 2-ketogluconate production ((**c**) & (**d**)), as well as formation of PCA ((**e**) & (**f**)). The reactors were actively aerated at 0.8 L/min for the first 24 h of the experiment and afterwards operated under passive aeration (dotted line). Rhamnolipids could not be detected.

Table S1: Primers used for tailoring heterologous rhamnolipid-producing *P. putida* with phenazine production.

No	Primer	Sequence 5′	Function
1	*rhlAB*-f	GTACCGAATTCCTCGAGTGGG CTCAACCTGGGAACTG	amplifying the *rhlAB* genes from PAO1, containing a Xba1 restriction site with overlapping regions for Gibson assembly to the pJNN plasmid backbone
2	*rhlAB*-r	CCGACGTCGCATGCTCCTCAC CGCTACACAGGAAATTC	
3	*rhlAB.MS*-f	GTCTTTTTTCGGCCGCGTACCA GGAGGAGAGATG	amplifying the *rhlAB* genes from PAO1, containing a Xba1 restriction site with overlapping regions for Gibson assembly to the pJNN.*MS* plasmid backbone
4	*rhlAB.MS*-r	CTGGATCTGGCCTAGGACTCT AGAATTCAGGACGC	
5	CP.*rhlAB*-f	AGATGCGGCGCGAAAGTCTG	amplifying part of the *rhl*A gene up to the *nagR/pNagAa* promoter region of the pJNN plasmid backbone (colony PCR verification)
6	CP.*rhlAB*-r	CGCGCCTGCTCGTATTCGCC	
7	CP.*M+S.rhlAB*-f	CTAGGCCAGATCCAGCGG	amplifying part of the *rhl*A gene up to the *nagR/pNagAa* promoter region of the pJNN.*MS* plasmid backbone (colony PCR verification)
8	CP.*M+S.rhlAB*-r	GCGGCCGAAAAAAGACCCGC	
9	Seq_*rhlA*	TGGCCGAACATTTCAACGTG	sequencing of the *rhlA* gene
10	Seq_*rhlB*	CTGTTCGACGGCAGTATCCC	sequencing the *rhlB* gene
